# A gene signature can predict risk of MGUS progressing to multiple myeloma

**DOI:** 10.1186/s13045-023-01472-y

**Published:** 2023-06-29

**Authors:** Fumou Sun, Yan Cheng, Jun Ying, David Mery, Samer Al Hadidi, Visanu Wanchai, Eric R. Siegel, Hongwei Xu, Dongzheng Gai, Timothy Cody Ashby, Clyde Bailey, Jin-Ran Chen, Carolina Schinke, Sharmilan Thanendrarajan, Maurizio Zangari, Siegfried Janz, Bart Barlogie, Frits Van Rhee, Guido Tricot, John D. Shaughnessy, Fenghuang Zhan

**Affiliations:** 1 Myeloma Center, Department of Internal Medicine, Winthrop P. Rockefeller Cancer Institute, University of Arkansas for Medical Sciences, 4301 W. Markham St. Slot# 508, Little Rock, AR 72205 USA; 2 Department of Biostatistics, University of Arkansas for Medical Sciences, Little Rock, AR USA; 3 Department of Biomedical Informatics, University of Arkansas for Medical Sciences, Little Rock, AR USA; 4 Arkansas Children’s Nutrition Center, University of Arkansas for Medical Sciences, Little Rock, AR USA; 5 Division of Hematology and Oncology, Department of Medicine, Medical College of Wisconsin, Milwaukee, WI USA

**Keywords:** Multiple myeloma, Monoclonal gammopathy of undetermined significance (MGUS), Gene expression profiling, Gene signature, Prediction model

## Abstract

Multiple myeloma is preceded by monoclonal gammopathy of undetermined significance (MGUS). Serum markers are currently used to stratify MGUS patients into clinical risk groups. A molecular signature predicting MGUS progression has not been produced. We have explored the use of gene expression profiling to risk-stratify MGUS and developed an optimized signature based on large samples with long-term follow-up. Microarrays of plasma cell mRNA from 334 MGUS with stable disease and 40 MGUS that progressed to MM within 10 years, was used to define a molecular signature of MGUS risk. After a three-fold cross-validation analysis, the top thirty-six genes that appeared in each validation and maximized the concordance between risk score and MGUS progression were included in the gene signature (GS36). The GS36 accurately predicted MGUS progression (C-statistic is 0.928). An optimal cut-point for risk of progression by the GS36 score was found to be 0.7, which identified a subset of 61 patients with a 10-year progression probability of 54.1%. The remainder of the 313 patients had a probability of progression of only 2.2%. The sensitivity and specificity were 82.5% and 91.6%. Furthermore, combination of GS36, free light chain ratio and immunoparesis identified a subset of MGUS patients with 82.4% risk of progression to MM within 10 years. A gene expression signature combined with serum markers created a highly robust model for predicting risk of MGUS progression. These findings strongly support the inclusion of genomic analysis in the management of MGUS to identify patients who may benefit from more frequent monitoring.

**Supplementary Information:**

The online version contains supplementary material available at 10.1186/s13045-023-01472-y.

## To the editor

Multiple myeloma (MM) is preceded by monoclonal gammopathy of undetermined significance (MGUS) [1, 2] with a 1.0% annual risk of MGUS progressing to MM [3]. MM patients with a prior known MGUS have better overall survival than those without prior MGUS [4]. At present, using serum markers, MGUS patients can be stratified into clinical risk groups that estimate the risk of progression from MGUS to MM, leading to the development of clinical consensus guidelines [5, 6]. Recently, the Dana-Farber Cancer Institute established the PANGEA model, which included bone marrow plasma cell percentage (BMPC%) in the prediction of myeloma progression, improving the accuracy of prediction [7]. They do not incorporate genetic or whole genome characteristics. All genetic changes associated with myelomagenesis are also present at the MGUS stage [8]. A robust high-risk molecular signature predicting MGUS progression is lacking.

**Fig. 1:**
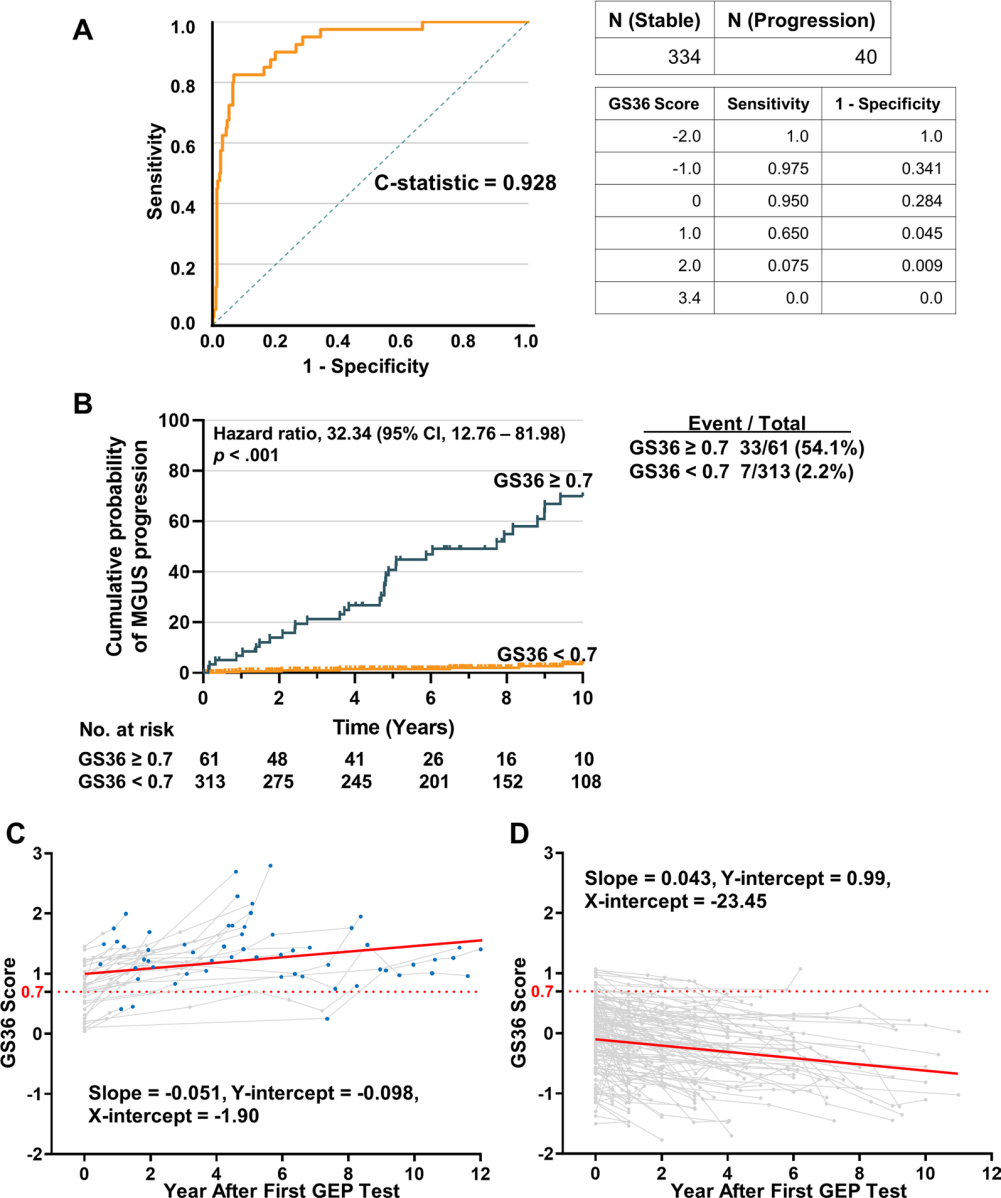
GS36 predicted MGUS progression. (**A**) MGUS Time-to-progression Curves in the GS36. Receiver operating characteristic curve (ROC) based on GS36. The C-statistic of ROC is 0.928. (**B**) Time-to-progression curve based on GS36. Among 40 patients who developed MM in 10 years, 33 patients had a GS36 ≥ 0.7 or test positive (T+), yielding a sensitivity of 82.5%. For the remaining 334 stable patients, only 28 patients had GS36 ≥ 0.7, yielding a false positive rate of 8.4% or a specificity of 91.6%. Also, 61 patients were identified to have T+ , while the remaining 313 patients tested negative (T−) or had their GS36 < 0.7. Among the 61 T+ patients, 33 patients progressed to MM in 10 years, yielding a positive predictive value (PPV) of 54.1%. For the 313 T− patients, only 7 patients developed MM in 10 years, yielding a probability of MM progression of 2.2%, or a negative predictive value (NPV) of 97.8%. (**C**) A total of 593 serial microarray analyses were performed on CD138-selected bone marrow samples from 174 MGUS cases with a baseline sample. The GS36 score invariably increased as the patients transitioned from MGUS into MM. (**D**) The GS36 score decreased in the group without progression to MM. Each grey line in the plot represents a unique patient and each point represents a unique GS36 score at that time. Blue point represents the patient at MM stage. Red line is the general trend line

To address this gap, 374 consecutive MGUS patients with baseline gene expression profiling (GEP) data were enrolled to establish the risk model. Of these, 40 patients progressed to MM within 10 years (progressing group), while 334 MGUS had not progressed (stable group). The clinical characteristics of the cohorts are shown in Additional file 2: Table S1. Microarray analysis was performed on mRNA from patients’ plasma cells. We used three-fold cross-validation to identify the gene list. The top thirty-six genes that appeared in each validation and maximized the concordance between risk score and MGUS progression were included in the “gene signature 36” (GS36) (Additional file 2: Table S2). The GS36 score accurately predicted MGUS progression (Harrell's C-statistic is 0.928) (Fig. 1A). An optimal cut-point for risk of progression by the GS36 score was 0.7. Among 40 progressing patients, 33 patients had a GS36 ≥ 0.7, yielding a sensitivity of 82.5%. For the 334 stable patients, only 28 patients had GS36 ≥ 0.7, yielding a specificity of 91.6%. Among the 61 GS36 ≥ 0.7 patients, 33 patients progressed to MM, yielding a probability of MM progression of 54.1%. For the 313 GS36 < 0.7 patients, only 7 patients developed MM, yielding a probability of MM progression of 2.2% (Fig. 1B). Details of methods are provided in Additional file 3.

Comparison with GEP70 showed that GS36 was better at predicting progression (Additional file 1: Fig. S1). Supervised clustering revealed that 24 genes were down-regulated and 12 up-regulated in high-risk MGUS cell samples (Additional file 1: Fig.S2). A total of 593 serial microarrays from 174 MGUS cases revealed that the GS36 score invariably increased as the patients transitioned from MGUS into MM, while the score decreased in the non-progressing group (Fig. 1C, D). We performed external validation using an independent dataset from patients enrolled on SWOG-S0120 [9]. In this dataset, 3 patients progressed to MM, while the other 54 MGUS had not progressed. Among 3 patients who developed MM, all had a GS36 ≥ 0.7. For the remaining 54 stable patients, only 4 patients had GS36 ≥ 0.7, yielding a probability of MM progression of 7.4% (Additional file 1: Fig.S3).

Clinical characteristics were assessed for their association with time to MM progression using a Cox proportional hazard (CPH) model (Additional file 2: Table S3). Increased risk of progression to MM was associated with bone marrow plasma cell (BMPC)% ≥ 7.5%, M-protein ≥ 1.5 g/dL, abnormal free light chains (FLC) ratio (< 0.1 or > 10) and decreased uninvolved immunoglobulins (DUIg). In addition to the hazard ratio (HR) for GS36 (HR = 33.72, *p* < 0.001) being very high, DUIg (HR = 3.67, *p* = 0.002) and abnormal FLC ratio (HR = 3.07, *p* = 0.001) were significant characteristics. The significant characteristics in the univariate CPH models were included in a multivariate CPH model. The results demonstrated that GS36 (HR = 31.32, *p* < 0.001), DUIg (HR = 2.76, *p* = 0.021) and abnormal FLC ratio (HR = 2.32, *p* = 0.022) are independent risk factors for progression (Additional file 2: Table S4).

**Fig. 2:**
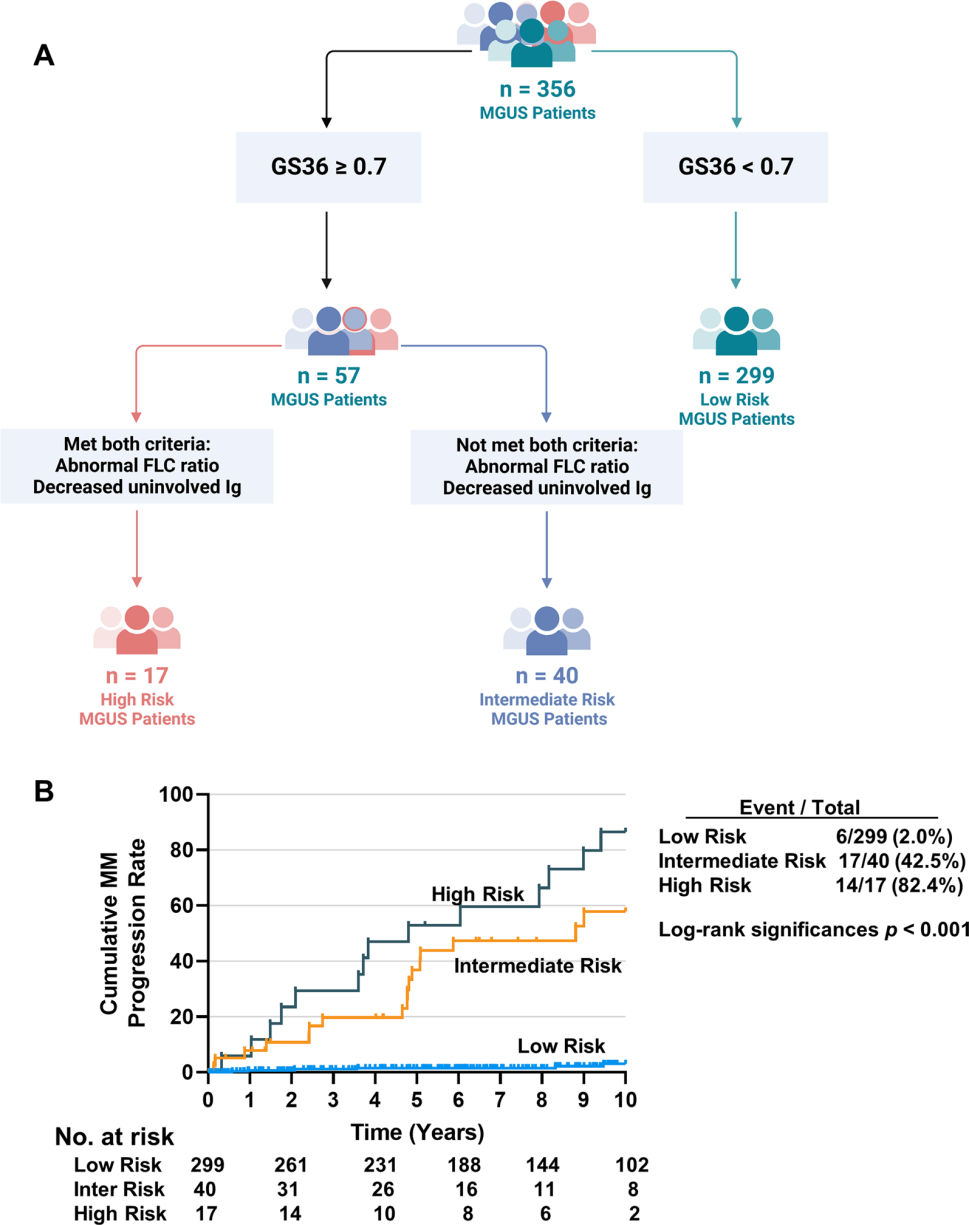
Recursive partitioning for MGUS progression. (**A**) A recursive partitioning algorithm used GS36 and clinical variables to define distinct clinically relevant risk groups. The low-risk MGUS patients were defined according to the following criteria: GS36 < 0.7. The high-risk MGUS patients were characterized as: GS36 ≥ 0.7, abnormal FLC ratio and decreased uninvolved immunoglobulins. An intermediate-risk group had a GS36 ≥ 0.7 but had an abnormal FLC ratio or decreased uninvolved immunoglobulins. (**B**) To test the accuracy of the UAMS MGUS risk model, we performed Kaplan–Meier analysis to identify the 10-year progression probability. The predicted values (or probabilities) of MGUS progression were 2.0%, 42.5% and 82.4% in the low-, intermediate-, and high-risk groups respectively

We combined clinical and molecular variables to model high-risk MGUS using recursive partitioning. The high-risk MGUS patients were characterized as: GS36 ≥ 0.7, abnormal FLC ratio and DUIg (Fig. 2A). This was termed the UAMS risk model. The 10-year probabilities of MGUS progression were 2.0% and 82.4% in the low- and high-risk groups (Fig. 2B).

In a comparison of the UAMS model with Mayo Clinic model [10] and Memorial Sloan Kettering Cancer Center (MSK) model [11], the UAMS model shows great promise, especially considering the SWOG-S0120 data presented (Additional file 2: Table S5 and Additional file 1: Fig. S4 A, B). However, comprehensive external validation using additional patient cohorts will be necessary to accurately assess the performance of the UAMS model in comparison to established models. The Venn diagram depicted in Additional file 1: Fig. S4C showed the overlapping quantities of the patients considered high-risk by UAMS, MSK, and Mayo models. High-risk was diagnosed across all definitions in 8 patients (15.4%).

The top-ranked genes in the GS36 are immunoglobulins (Ig) genes (*IGLV1-44, IGKC, IGHA1*), which are significantly decreased in the high-risk MGUS group. GEP of samples with a high percentage of normal plasma cells will produce signals for numerous immunoglobulin genes. We are of the opinion that the high levels of Ig genes reflect the presence of normal PC in the purified sample. Thus, our data imply that the absence of a normal plasma cell signature in MGUS is associated with early progression.

In conclusion, we have generated a novel model that integrates molecular and clinical variables that can significantly improve the predictability of progression to overt MM.

## Supplementary Information

**Additional file 1.** Supplementary Figures.

**Additional file 2.** Supplementary Tables.

**Additional file 3.** Supplementary Methods.

## Data Availability

All data generated or analyzed during this study are included in this published article.

## Abbreviations

MM: Multiple myeloma
MGUS: Monoclonal gammopathy of undetermined significance
GS36: Gene signature 36
GEP: Gene expression profiling
ROC: Receiver operating characteristic
GEP70: 70-Gene signature
FLC: Free light chain
PPV: Positive predictive value
NPV: Negative predictive value
CPH: Univariate Cox proportional hazard
DUIg: Decreased uninvolved immunoglobulin
BMPC: Bone marrow plasma cell
MSK: Memorial Sloan Kettering Cancer Center
