# Supplementary material for: A gene signature can predict risk of MGUS progressing to multiple myeloma
Source: J Hematol Oncol. 2023 Jun 29;16:70. doi: 10.1186/s13045-023-01472-y (PMC10308756; doi:10.1186/s13045-023-01472-y)

Additional file 1: Supplementary Figures

Table of Contents

| Supplement | Title | page |
| --- | --- | --- |
| Figure S1 | ROC Curve and MGUS Time-to-progression Curves based on GEP70 | 2 |
| Figure S2 | The heatmap of the GS36 genes expression profiles | 3 |
| Figure S3 | External validation using an independent datasets SWOG-S0120 | 4 |
| Figure S4 | MGUS Time-To-Progression Comparison on Mayo Risk Model and Memorial Sloan Kettering Cancer Center (MSK) Risk Model | 5 |

**Figure S1. MGUS Time-to-progression Curves based on GEP70.** (A). Receiver operating characteristic curve (ROC) based on GEP70. The C-statistic of ROC is 0.626; (B) Time-to-progression curve based on GEP70.


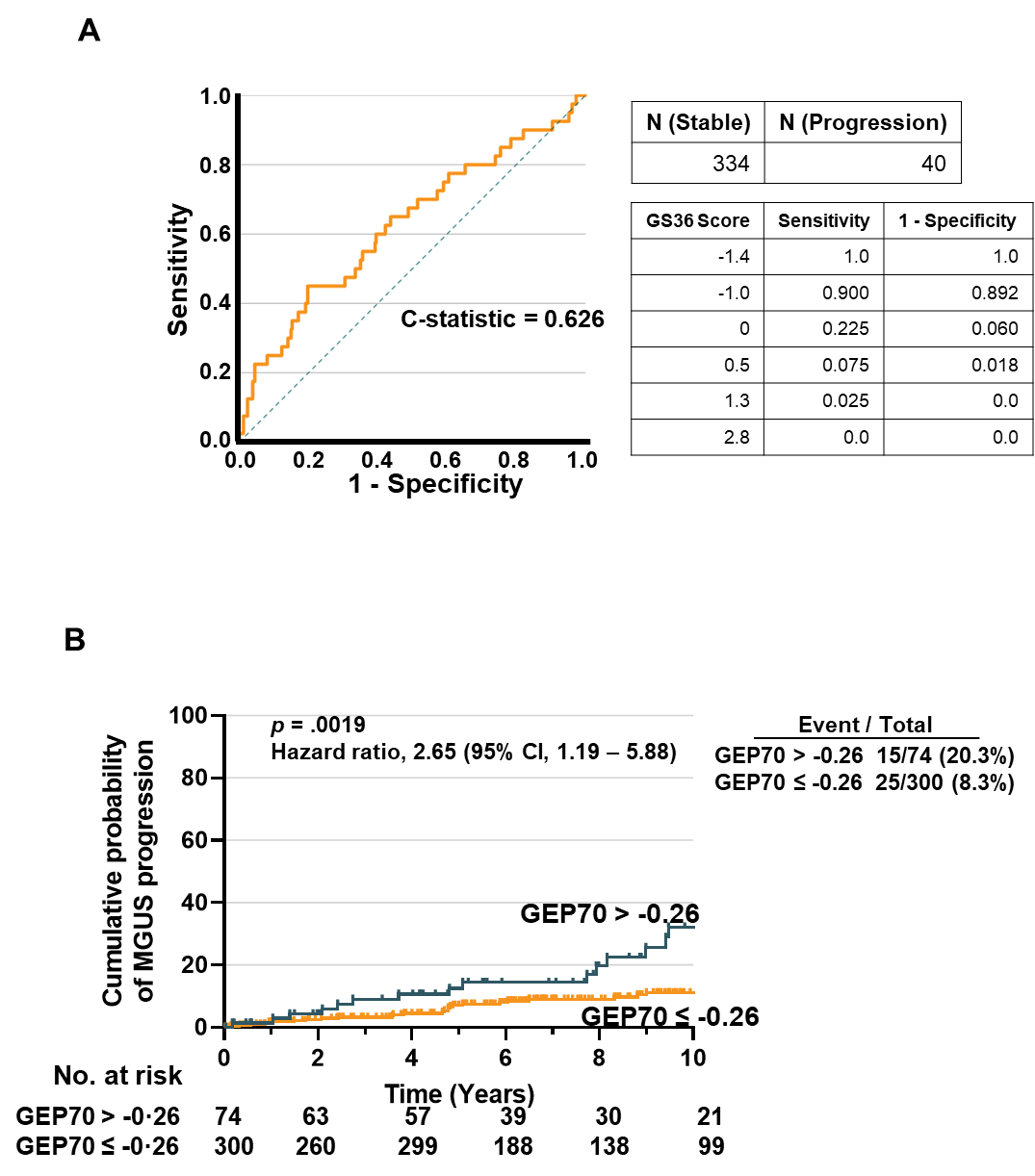


**Figure S2. The heatmap of the GS36 genes expression profiles** (red, relative high expression; blue, relative low expression) between stable and progressing risk groups.


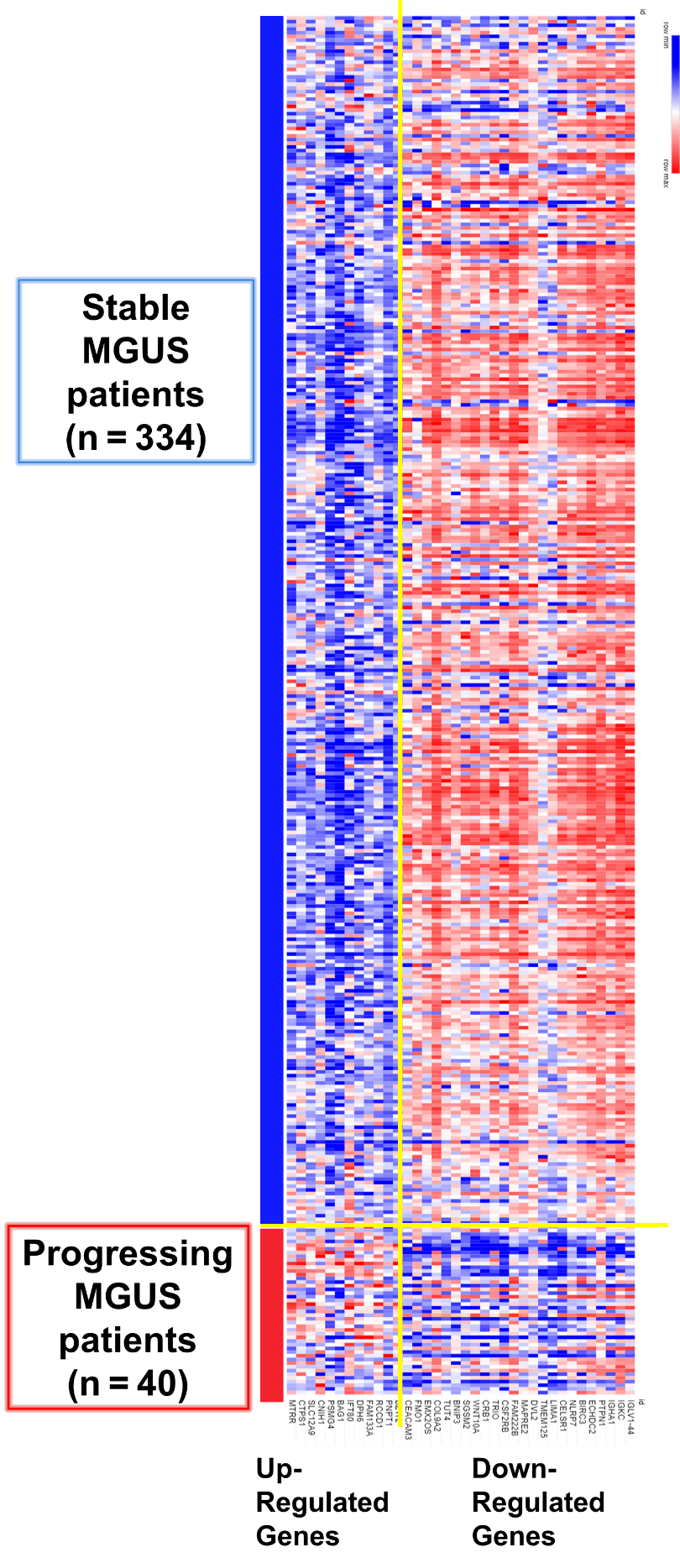


**Figure S3. External validation using an independent datasets SWOG-S0120.** (A) The scatter plot of GS36 score in SWOG-S0120 datasets. Three patients progressed to MM within 10 years, while the other 54 MGUS had not progressed. Among three patients who developed MM, all three had a GS36 ≥ 0.7. For the remaining 54 stable patients, only 4 patients had GS36 ≥ 0.7. (B) Time-to-progression curve of SWOG-S0120 datasets based on GS36.


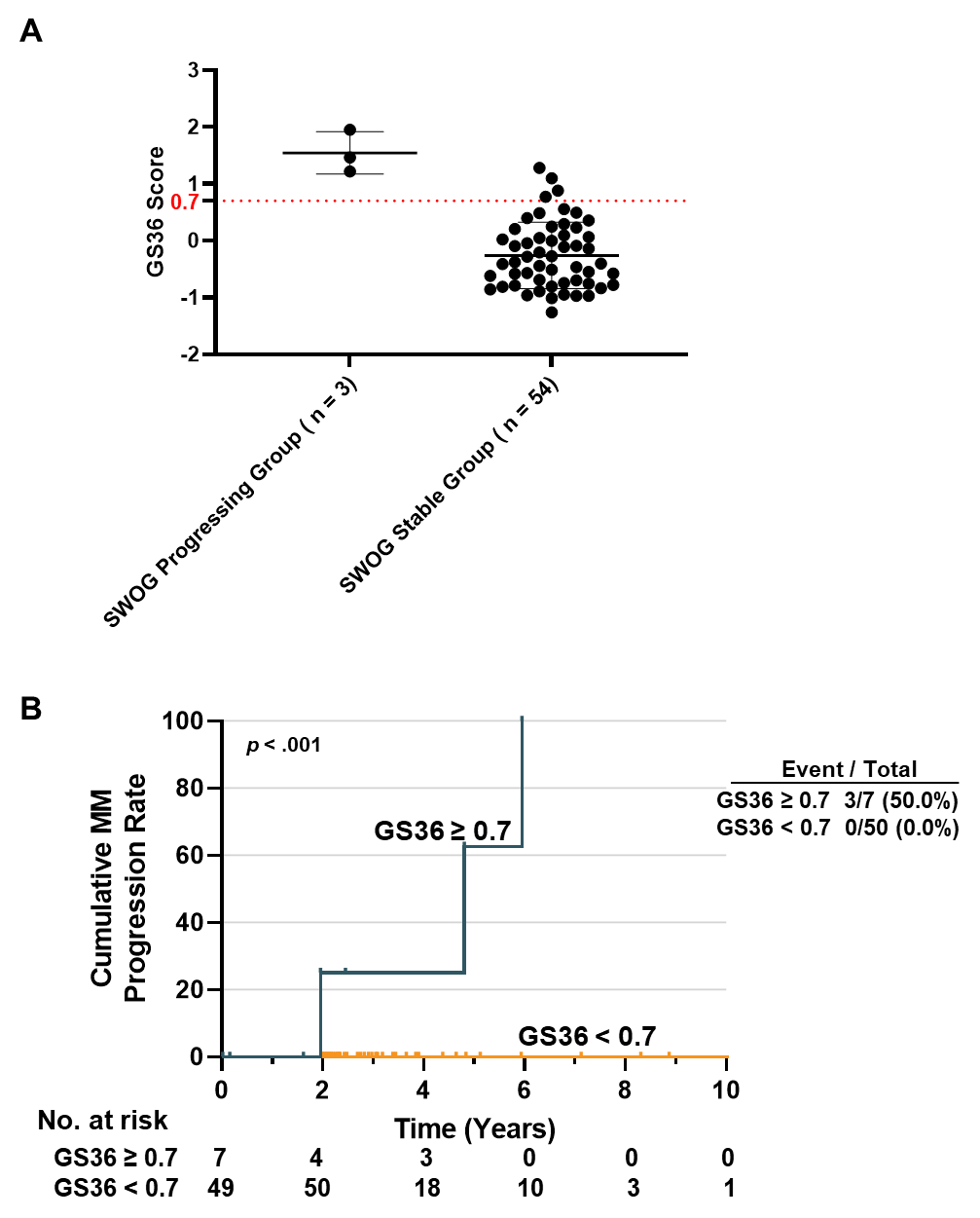


**Figure S4. MGUS Time-To-Progression Comparison on Mayo Risk Model and Memorial Sloan Kettering Cancer Center (MSK) Risk Model.** (A) 10-year-progression curve based on Mayo risk model. (B) 10-year-progression curve based on MSK risk model. (C) Venn diagram depicting the overlapping quantities of the patients considered high-risk by UAMS, MSK, and Mayo models. The green panel represents MSK model; the blue panel represents Mayo model; the red panel represents UAMS model.


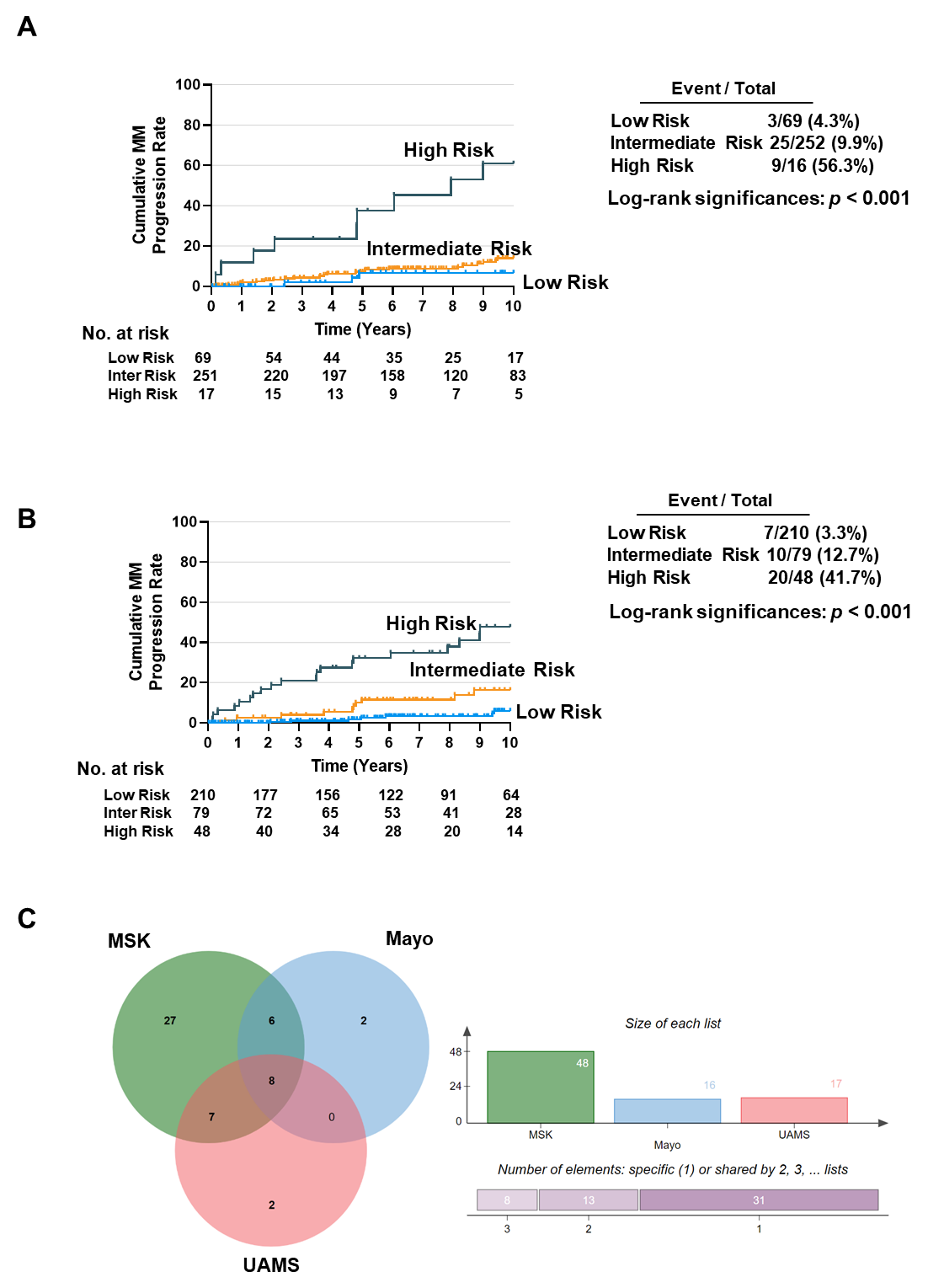

Supplement: Supplementary file 1 — Additional file 1. Supplementary Figures. [file 13045_2023_1472_MOESM1_ESM.docx]
