# Supplementary material for: A gene signature can predict risk of MGUS progressing to multiple myeloma
Source: J Hematol Oncol. 2023 Jun 29;16:70. doi: 10.1186/s13045-023-01472-y (PMC10308756; doi:10.1186/s13045-023-01472-y)
Supplement: Supplementary file 2 — Additional file 2. Supplementary Tables. [file 13045_2023_1472_MOESM2_ESM.docx]

Additional file 2: Supplementary Tables

Table of Contents

| Supplement | Title | page |
| --- | --- | --- |
| Table S1 | Summary of Demographics and Baseline Clinical Characteristics of MGUS Patients | 2 |
| Table S2 | Gene Signature 36 (GS36) Associated with MGUS Progression | 3 |
| Table S3 | Univariate Cox Proportional Hazards Analysis of Risk Factors of MGUS Progression | 4 |
| Table S4 | Hazard Ratios (HRs) of MGUS Progression Predicted by Risk Factors Using a Multivariate Cox Proportional Hazard Model | 5 |
| Table S5 | Comparison of the UAMS, Mayo and MSK MGUS Risk Model | 6 |

**Table S1. Summary of Demographics and Baseline Clinical Characteristics of MGUS Patients**

| **Characteristic** | **Combined** | **Progressing group** | **Stable group** | ***P*-Value** |
| --- | --- | --- | --- | --- |
|  | n of patients in the category / n of observed patients (%) | | |  |
| Age ≥ 65 year | 164/374 (44%) | 18/40 (45%) | 146/334 (44%) | 0.877 |
| Race | | | | |
| White | 306/374 (82%) | 35/40 (88%) | 271/334 (81%) | 0.324 |
| Black | 61/374 (16%) | 5/40 (13%) | 56/334 (17%) | 0.49 |
| Female | 189/374 (51%) | 20/40 (50%) | 169/334 (51%) | 0.943 |
| Kappa light chains | 233/374 (62%) | 25/40 (63%) | 208/334 (62%) | 0.978 |
| Lambda light chains | 123/374 (33%) | 15/40 (38%) | 108/334 (32%) | 0.511 |
| IgA subtype | 52/374 (14%) | 5/40 (13%) | 47/334 (14%) | 0.786 |
| IgG subtype | 276/374 (74%) | 35/40 (88%) | 241/334 (72%) | 0.037 |
| BMPC% ≥ 7.5% | 166/367 (45%) | 28/40 (70%) | 138/327 (42%) | < 0.001 |
| Albumin < 3.5 g/dl | 60/371 (16%) | 5/40 (13%) | 55/331 (17%) | 0.504 |
| Beta2-microglobulin ≥ 4 mg/liter | 49/361 (14%) | 9/39 (23%) | 40/322 (12%) | 0.067 |
| Creatinine = 1.1 - 1.9 mg/dl | 136/373 (36%) | 20/40 (50%) | 116/333 (35%) | 0.06 |
| Hemoglobin = 10 - 12 g/dl | 77/372 (21%) | 9/40 (23%) | 68/332 (20%) | 0.766 |
| Platelets ≤ 150 × 10^9^/L | 41/372 (11%) | 3/40 (7.5%) | 38/332 (11%) | 0.598 ^a^ |
| Lactate dehydrogenase ≥ 190 IU/liter | 54/370 (15%) | 8/40 (20%) | 46/330 (14%) | 0.305 |
| Serum M-protein ≥ 1.5 g/dL | 106/352 (30%) | 23/40 (58%) | 83/312 (27%) | < 0.001 |
| Abnormal FLC ratio (<0.26 or >1.65) | 229/356 (64%) | 26/37 (70%) | 203/319 (64%) | 0.425 |
| Abnormal FLC ratio (<0.1 or >10) | 74/356 (21%) | 16/37 (32%) | 58/319 (18%) | < 0.001 |
| Decreased uninvolved immunoglobulins (DUIg) ^b^ | 202/374 (54%) | 33/40 (83%) | 169/334 (51%) | < 0.001 |
| GEP seven molecular MM subgroups | | | | |
| CD-1 | 16/374 (4.3%) | 3/40 (7.5%) | 13/334 (3.9%) | 0.396 ^a^ |
| CD-2 | 93/374 (25%) | 11/40 (28%) | 82/334 (25%) | 0.683 |
| HY | 64/374 (17%) | 14/40 (35%) | 50/334 (15%) | 0.001 |
| LB | 89/374 (24%) | 3/40 (7.5%) | 87/334 (26%) | 0.01 |
| MF | 47/374 (13%) | 5/40 (13%) | 42/334 (13%) | 0.989 |
| MS | 14/374 (3.7%) | 2/40 (5%) | 12/334 (3.6%) | 0.653 ^a^ |
| PR | 6/374 (1.6%) | 1/40 (2.5%) | 5/334 (1.5%) | 0.495 ^a^ |
| Unknown | 45/374 (12%) | 1/40 (2.5%) | 44/334 (13%) | 0.067 ^a^ |
| GEP 70-gene risk > −0.26 | 74/374 (20%) | 15/40 (38%) | 59/334 (18%) | 0.003 |
| Abbreviations: MGUS, monoclonal gammopathy of undetermined significance; BMPC, bone marrow plasma cell; M-protein, monoclonal protein; FLC, free light chains; GEP, gene expression profiling; CD-1, cyclin D-1; CD-2, cyclin D-2; C; MF, MAF; MS, multiple myeloma-SET; PR, proliferation.  ^a^ P-Value computed using Fisher's exact test.  ^b^ Decreased uninvolved immunoglobulins: <600 mg/dL if IgG, <50 mg/dL if IgM, <100 mg/dL if IgA. | | | | |

**Table S2. Gene Signature 36 (GS36) Associated with MGUS Progression**

|  | **Probeset** | **Gene** | **Location** | **Q-Value** |
| --- | --- | --- | --- | --- |
| Down-Regulated Genes | 234764_x_at | IGLV1-44 | chr22q11.22 | 3.10E-09 |
|  | 211835_at | IGKC | chr2p11.2 | 5.52E-09 |
|  | 1561937_x_at | IGHA1 | chr14q32.33 | 4.43E-08 |
|  | 202716_at | PTPN1 | chr20q13.13 | 1.87E-07 |
|  | 235305_s_at | ECHDC2 | chr1p32.3 | 6.47E-07 |
|  | 210538_s_at | BIRC3 | chr11q22.2 | 1.08E-06 |
|  | 237461_at | NLRP7 | chr19q13.42 | 1.27E-06 |
|  | 41660_at | CELSR1 | chr22q13.31 | 3.47E-06 |
|  | 217892_s_at | LIMA1 | chr12q13.12 | 4.17E-05 |
|  | 225822_at | TMEM125 | chr1p34.2 | 7.08E-05 |
|  | 57532_at | DVL2 | chr17p13.1 | 7.92E-05 |
|  | 213489_at | MAPRE2 | chr18q12.1 | 8.48E-05 |
|  | 222641_s_at | FAM222B | chr17q11.2 | 8.51E-05 |
|  | 205159_at | CSF2RB | chr22q12.3 | 9.60E-05 |
|  | 209012_at | TRIO | chr5p15.2 | 9.95E-05 |
|  | 220522_at | CRB1 | chr1q31.3 | 1.20E-04 |
|  | 223709_s_at | WNT10A | chr2q35 | 3.28E-04 |
|  | 36129_at | SGSM2 | chr17p13.3 | 3.29E-04 |
|  | 201848_s_at | BNIP3 | chr10q26.3 | 3.86E-04 |
|  | 212704_at | TUT4 | chr1p32.3 | 3.94E-04 |
|  | 213622_at | COL9A2 | chr1p34.2 | 4.38E-04 |
|  | 232531_at | EMX2OS | chr10q26.11 | 4.67E-04 |
|  | 205666_at | FMO1 | chr1q24.3 | 5.02E-04 |
|  | 210789_x_at | CEACAM3 | chr19q13.2 | 7.47E-04 |
| Up-Regulated Genes | 217809_at | BZW2 | chr7p21.1 | 1.56E-06 |
|  | 225291_at | PNPT1 | chr2p16.1 | 1.53E-05 |
|  | 226488_at | RCCD1 | chr15q26.1 | 1.61E-05 |
|  | 231131_at | FAM133A | chrXq21.32 | 4.41E-05 |
|  | 238662_at | DPH6 | chr15q14 | 8.09E-05 |
|  | 226098_at | IFT80 | chr3q25.33 | 1.74E-04 |
|  | 202387_at | BAG1 | chr9p13.3 | 3.43E-04 |
|  | 228217_s_at | PSMG4 | chr6p25.2 | 5.81E-04 |
|  | 225553_at | CNIH1 | chr14q22.2 | 5.83E-04 |
|  | 223995_at | SLC12A9 | chr7q22.1 | 7.21E-04 |
|  | 202613_at | CTPS1 | chr1p34.2 | 7.72E-04 |
|  | 203200_s_at | MTRR | chr5p15.31 | 7.92E-04 |
| Abbreviations: MGUS, monoclonal gammopathy of undetermined significance. | | | | |

**Table S3. Univariate Cox Proportional Hazards Analysis of Risk Factors of MGUS Progression**

| **Characteristic** | **n of patients in the category /**  **n of observed patients (%)** | **HR (95% CI)** | **P-Value** |
| --- | --- | --- | --- |
| GS36 ≥ 0.7 | 61/374 (16%) | 33.72 (14.86, 76.53) | < 0.001 |
| Age ≥65 year | 164/374 (44%) | 1.05 (0.56, 1.96) | 0.871 |
| Race | | | |
| White | 306/374 (82%) | 1.64 (0.64, 4.19) | 0.302 |
| Black | 61/374 (16%) | 0.68 (0.27, 1.74) | 0.425 |
| Female | 189/374 (51%) | 0.97 (0.52, 1.80) | 0.918 |
| Kappa light chains | 233/374 (62%) | 1.01 (0.53, 1.92) | 0.976 |
| Lambda light chains | 123/374 (33%) | 1.21 (0.64, 2.30) | 0.558 |
| IgA subtype | 52/374 (14%) | 0.81 (0.32, 2.06) | 0.654 |
| IgG subtype | 276/374 (74%) | 2.45 (0.96, 6.25) | 0.061 |
| BMPC% ≥ 7.5% | 166/367 (45%) | 2.54 (1.29, 4.99) | 0.007 |
| Albumin < 3.5 g/dl | 60/371 (16%) | 0.73 (0.29, 1.87) | 0.513 |
| Beta2-microglobulin ≥ 4 mg/liter | 49/361 (14%) | 1.96 (0.93, 4.13) | 0.078 |
| Creatinine = 1.1 - 1.9 mg/dl | 136/373 (36%) | 1.86 (1.00, 3.46) | 0.05 |
| Hemoglobin = 10 - 12 g/dl | 77/372 (21%) | 1.06 (0.50, 2.22) | 0.882 |
| Platelets ≤ 150 × 10^9^/L | 41/372 (11%) | 0.68 (0.21, 2.19) | 0.513 |
| Lactate dehydrogenase ≥ 190 IU/liter | 54/370 (15%) | 1.52 (0.70, 3.31) | 0.287 |
| Serum M-protein ≥ 1.5 g/dL | 106/352 (30%) | 2.84 (1.52, 5.32) | 0.001 |
| Abnormal FLC ratio (<0.26 or >1.65) | 229/356 (64%) | 1.23 (0.61, 2.48) | 0.572 |
| Abnormal FLC ratio (<0.1 or >10) | 76/356 (21%) | 3.07 (1.60, 5.89) | 0.001 |
| Decreased uninvolved immunoglobulins (DUIg) ^a^ | 202/374 (54%) | 3.67 (1.62, 8.30) | 0.002 |
| GEP seven molecular MM subgroups | |  |  |
| CD-1 | 16/374 (4.3%) | 1.75 (0.54, 5.68) | 0.352 |
| CD-2 | 93/374 (25%) | 1.30 (0.65, 2.60) | 0.457 |
| HY | 64/374 (17%) | 2.61 (1.36, 5.00) | 0.004 |
| LB | 89/374 (24%) | 0.33 (0.12, 0.92) | 0.033 |
| MF | 47/374 (13%) | 0.96 (0.38, 2.45) | 0.929 |
| MS | 14/374 (3.7%) | 1.37 (0.33, 5.67) | 0.667 |
| PR | 6/374 (1.6%) | 1.43 (0.20, 10.41) | 0.725 |
| Unknown | 45/374 (12%) | 0.19 (0.03, 1.36) | 0.098 |
| Abbreviations: MGUS, monoclonal gammopathy of undetermined significance; HR, hazard ratio; CI, confidence interval; GS36, gene signature 36; BMPC, bone marrow plasma cell; M-protein, monoclonal protein; FLC, free light chains; GEP, gene expression profiling; CD-1, cyclin D-1; CD-2, cyclin D-2; C; MF, MAF; MS, multiple myeloma-SET; PR, proliferation.  ^a^ Decreased uninvolved immunoglobulins: <600 mg/dL if IgG, <50 mg/dL if IgM, <100 mg/dL if IgA. | | | |

**Table S4. Hazard Ratios (HRs) of MGUS Progression Predicted by Risk Factors Using a Multivariate Cox Proportional Hazard Model**

| **Risk factor** | **n of patients in the category /n of observed patients (%)** | **HR (95% CI)** | ***P*-Value** |
| --- | --- | --- | --- |
| GS36 ≥ 0.7 | 61/374 (16%) | 31.32 (12.70, 77.26) | < 0.001 |
| BMPC% ≥ 7.5% | 166/367 (45%) | 1.19 (0.55, 2.56) | 0.662 |
| Serum M-protein ≥ 1.5 g/dL | 106/352 (30%) | 0.79 (0.38, 1.67) | 0.54 |
| Abnormal FLC ratio (<0.1 or >10) | 76/356 (21%) | 2.32 (1.13, 4.79) | 0.022 |
| Decreased uninvolved immunoglobulins (DUIg) ^a^ | 202/374 (54%) | 2.76 (1.16, 6.55) | 0.021 |
| GEP HY subgroup | 64/374 (17%) | 1.46 (0.71, 3.00) | 0.306 |
| GEP LB subgroup | 89/374 (24%) | 0.43 (0.12, 1.50) | 0.185 |
| Abbreviations: MGUS, monoclonal gammopathy of undetermined significance; HR, hazard ratio; CI, confidence interval; GS36, gene signature 36; BMPC, bone marrow plasma cell; M-protein, monoclonal protein; FLC, free light chains; GEP, gene expression profiling; HY, hyperdiploid; LB, low bone.  ^a^ Decreased uninvolved immunoglobulins: <600 mg/dL if IgG, <50 mg/dL if IgM, <100 mg/dL if IgA. | | | |

**Table S5. Comparison of the UAMS, Mayo and MSK MGUS Risk Model**

| **Models** | **Factors** | **Harrell's C-statistic (95% CI)** | **10-Year-Progression Probability** |
| --- | --- | --- | --- |
| UAMS Risk Model | GS36 ≥ 0  Abnormal FLC ratio (<0.1 or >10)  Decreased uninvolved immunoglobulins (DUIg) ^a^ | 0.89 (0.818, 0.961) | Low risk: 2.0%  Intermediate risk: 42.5%  High risk: 82.4% |
| Mayo Risk Model | M-protein ≥ 1.5 g/dL  non-IgG MGUS  Abnormal FLC ratio (<0.26 or >1.65) | 0.653 (0.555, 0.752) | Low risk: 4.3%  Intermediate risk: 10%  High risk: 56% |
| MSK Risk Model | MGUS:  IgA MGUS  M-protein ≥ 1.5 g/dL  Abnormal FLC ratio (<0.1 or >10)  Decreased uninvolved immunoglobulins (DUIg) ^a^ | 0.79 (0.704, 0.877) | Low risk: 3.3%  Intermediate risk: 13%  High risk: 42% |
|  | Light Chain MGUS:  Abnormal FLC ratio (<0.1 or >10)  Decreased uninvolved immunoglobulins (DUIg) ^a^ |  |  |
| Abbreviations: MGUS, monoclonal gammopathy of undetermined significance; GS36, gene signature 36; M-protein, monoclonal protein; FLC, free light chains; UAMS, university of Arkansas for medical sciences; MSK, Memorial Sloan Kettering cancer center.  ^a^ Decreased uninvolved immunoglobulins: <600 mg/dL if IgG, <50 mg/dL if IgM, <100 mg/dL if IgA. | | | |
