# Supplementary material for: A gene signature can predict risk of MGUS progressing to multiple myeloma
Source: J Hematol Oncol. 2023 Jun 29;16:70. doi: 10.1186/s13045-023-01472-y (PMC10308756; doi:10.1186/s13045-023-01472-y)
Supplement: Supplementary file 3 — Additional file 3. Supplementary Methods. [file 13045_2023_1472_MOESM3_ESM.docx]

Additional file 3: Supplementary Methods

**Study Population and Eligibility Criteria**

GEP of highly purified bone marrow plasma cells was performed in 374 patients with MGUS. Of these, 174 patients had 593 GEPs on serial bone marrow samples. The institutional review board of the University of Arkansas for Medical Sciences approved the research studies, and all subjects provided written informed consent for sample procurement in accordance with the Declaration of Helsinki.

Patients had detailed clinical testing during follow-up, including hemogram and serum electrophoresis to quantify serum-M concentration, serum lactate dehydrogenase, serum β-2-microglobulin (B2M), and serum free light chain (SFLC).

Of the 374 MGUS cases, 15 lacked FLC ratio data, 19 M-protein information, and 3 lacked both the FLC ratio data and M-protein information.

**Plasma Cell Selection and Microarray Processing**

Plasma cells were enriched by anti-CD138 immunomagnetic bead selection of mononuclear cell fractions of bone marrow aspirates in a central laboratory. All samples applied to microarray contained more than 85% plasma cells as determined by 2-color flow cytometry (CD38^+^ and CD45^–/dim^) performed after selection. Total RNA was isolated with RNeasy Mini Kit (Qiagen, Valencia, CA). Double-stranded cDNA is synthesized from 6 ug total RNA. An in vitro transcription (IVT) reaction is then done to produce biotin-labeled cRNA from the cDNA. The cRNA is fragmented before hybridization. The protocol according to the manufacturer's instructions Expression Analysis Technical Manual, 2001, Affymetrix. Samples were hybridized to an Affymetrix U133Plus2.0 microarray according to the manufacturer's recommendations and then read on a GeneChip Scanner 3000 (Affymetrix). Arrays were scaled to an average intensity of 1500 and analyzed independently. The data were processed using Microarray Suite version 5.0 (MAS 5.0) with Affymetrix default analysis settings and global scaling as the normalization method [1]. The average purity of plasma cells was 89.6% with a range from 85% to 100%.

**Development of** **the Prediction Gene Signature**

GEPs were analyzed from 374 MGUS patients, 334 who remained stable, and 40 who progressed to MM, within 10 years of an MGUS diagnosis. Each Affymetrix signal was log2 transformed to correct the right skewness of its empirical distribution. Next, we used a 3-fold cross-validation method by randomly splitting patients into three datasets (folds): the first dataset included 124 individuals (111 stable and 13 progression); the second dataset included 125 individuals (111 stable and 14 progression); the third dataset including 125 individuals (112 stable and 13 progression). Each time one fold was called the test dataset while the remaining two folds were called the training dataset.

Each time one fold was called the test dataset while the remaining two folds were called the training dataset. In the training datasets, we compared the each of gene probes between the MGUS progression group and the stable group using t-tests and correcting for multiple comparisons through a Benjamini-Hochberg procedure. Then in the test datasets, we also compared the each of gene probes between the MGUS progression group and the stable group using t-tests and correcting for multiple comparisons through a Benjamini-Hochberg procedure. The gene probes with q-values < 0.05 in both training datasets and test datasets were treated as candidates. In the first validation process, there are 61 gene probes in both training datasets and test datasets; in the second validation process there are 46 gene probes in both; in the third validation process, there are 49 gene probes in both. In these GEPs, there are 36 gene probes that appeared in each validation and maximized the concordance between risk score and MGUS progression.

Furthermore, we conducted ROC analysis for the 36 genes identified and examined their performance in each validation. We found that these 36 genes consistently demonstrated stable ROC values across all validation iterations. The stable ROC values indicate that the predictive ability of these 36 genes remained consistent and reliable throughout the validation process. This finding provides further evidence of the robustness and stability of these genes in relation to the studied phenomenon.

A gene expression score was developed by subtracting the mean of down-regulated GEPs from the mean of up-regulated GEPs. The score’s performance in discriminating MGUS progression within 10 years was assessed the Harrell's concordance statistic (C-statistic) in the test dataset. Using an optimal cutoff that maximizes the average sensitivity and specificity on the ROC curve, we were able to further assess the accuracy of prediction sensitivity, specificity, positive predictive value (PPV) and negative predictive value (NPV) under the cutoff.

**Statistical Analysis**

Baseline characteristics of patients with MGUS were compared using Chi-square tests, or Fisher’s exact tests when fewer than 3 patients were observed in a categorical group. Time to MGUS progression was assessed by Kaplan-Meier curves and compared between groups using log-rank tests. Univariate Cox proportional hazard (CPH) models were used to assess individual risk factors in predicting the risk of MM progression. Significant risk factors from univariate CPH models were further assessed for their performance in predicting MGUS progression using a multivariate CPH model. An optimal cut-point for risk of progression by the gene score was obtained from X-tile bioinformatics software (Yale University, version 3.6.1). Statistical tests were performed with the software package SPSS 12.0 (SPSS, Chicago, IL).

**Data Availability**

The authors are committed to the open sharing of data. Raw data files for gene expression profiling data have been deposited in the Gene Expression Omnibus (https://www.ncbi.nlm.nih.gov/geo/, accession # is GSE235356) or contact corresponding authors.

**Code Sharing and Example Demonstration**

To facilitate the use of the GS36 score for evaluating risk stratification in MGUS, we will present an example code for processing a sample file. We will use the R programming language for the analysis.

For CSV file:

# Install and load the 'readr' package for CSV file import

install.packages("readr")

library(readr)

# Specify the file path of the CSV file containing gene expression data

csv_file <- "path/to/your/file.csv"

# Read the CSV file and store the data in a data frame

gene_expression_data <- read_csv(csv_file)

For CEL files (using the 'affy' package):

# Install and load the 'affy' package for CEL file import

install.packages("affy")

library(affy)

# Specify the directory path containing the CEL files

cel_directory <- "path/to/your/CEL/directory"

# Read the CEL files and preprocess the data using the robust multi-array average (RMA) method

cel_data <- ReadAffy(celfile.path = cel_directory)

gene_expression_data <- exprs(rma(cel_data))

# Perform a log2 transformation on the gene expression data

gene_expression_data <- log2(gene_expression_data)

# Specify the Probeset names of interest

probeset_names <- c("234764_x_at", "211835_at", "1561937_x_at", "202716_at", "235305_s_at", "210538_s_at", "237461_at", "41660_at", "217892_s_at", "225822_at", "57532_at", "213489_at", "222641_s_at", "205159_at", "209012_at", "220522_at", "223709_s_at", "36129_at", "201848_s_at", "212704_at", "213622_at", "232531_at", "205666_at", "210789_x_at", "217809_at", "225291_at", "226488_at", "231131_at", "238662_at", "226098_at", "202387_at", "228217_s_at", "225553_at", "223995_at", "202613_at", "203200_s_at")

# Extract the expression data for the specified Probesets

probeset_expression_data <- gene_expression_data[, probeset_names]

# Calculate the average expression for Down-Regulated Probesets

down_regulated <- rowMeans(probeset_expression_data[, c("234764_x_at", "211835_at", "1561937_x_at", "202716_at", "235305_s_at", "210538_s_at", "237461_at", "41660_at", "217892_s_at", "225822_at", "57532_at", "213489_at", "222641_s_at", "205159_at", "209012_at", "220522_at", "223709_s_at", "36129_at", "201848_s_at", "212704_at", "213622_at", "232531_at", "205666_at", "210789_x_at")])

# Calculate the average expression for Up-Regulated Probesets

up_regulated <- rowMeans(probeset_expression_data[, c("217809_at", "225291_at", "226488_at", "231131_at", "238662_at", "226098_at", "202387_at", "228217_s_at", "225553_at", "223995_at", "202613_at", "203200_s_at")])

# Subtract Down-Regulated from Up-Regulated

result <- up_regulated - down_regulated

# Classify samples as high-risk or low-risk MGUS based on the result

mgus_classification <- ifelse(result > 0.7, "High Risk MGUS", "Low Risk MGUS")

Please note that the code provided above is a general example and should be adjusted to suit your specific dataset and analysis requirements.

1. Zhan F, Huang Y, Colla S, Stewart JP, Hanamura I, Gupta S, et al. The molecular classification of multiple myeloma. Blood. 2006;108(6):2020-8; doi: 10.1182/blood-2005-11-013458.
